# Identification of host cellular proteins that interact with the M protein of a highly pathogenic porcine reproductive and respiratory syndrome virus vaccine strain

**DOI:** 10.1186/s12985-017-0700-1

**Published:** 2017-02-22

**Authors:** Qian Wang, Yanwei Li, Hong Dong, Li Wang, Jinmei Peng, Tongqing An, Xufu Yang, Zhijun Tian, Xuehui Cai

**Affiliations:** 1 Division of Swine Infectious Diseases, National Key Laboratory of Veterinary Biotechnology, Harbin Veterinary Research Institute, Chinese Academy of Agricultural Sciences, No.678, Haping street, Xiangfang District, Harbin, 150069 China; 2 National Engineering Research Center of Veterinary Biologics, Harbin, 150001 China; 3 Key Laboratory of Animal Epidemiology and Zoonosis of Ministry of Agriculture, College of Veterinary Medicine and State Key Laboratory of Agribiotechnology, China Agricultural University, Beijing, 100193 China; 4 North Guangdong Collaborative Innovation and Development Center of Pig Farming and Disease Control, Shaoguan University, Shaoguan, 512005 China

**Keywords:** HP-PRRSV, M protein, Host cellular proteins, Interaction, Bioinformatics

## Abstract

**Background:**

The highly pathogenic porcine reproductive and respiratory syndrome virus (HP-PRRSV) continues to pose one of the greatest threats to the swine industry. M protein is the most conserved and important structural protein of PRRSV. However, information about the host cellular proteins that interact with M protein remains limited.

**Methods:**

Host cellular proteins that interact with the M protein of HP-PRRSV were immunoprecipitated from MARC-145 cells infected with PRRSV HuN4-F112 using the M monoclonal antibody (mAb). The differentially expressed proteins were identified by LC-MS/MS. The screened proteins were used for bioinformatics analysis including Gene Ontology, the interaction network, and the enriched KEGG pathways. Some interested cellular proteins were validated to interact with M protein by CO-IP.

**Results:**

The PRRSV HuN4-F112 infection group had 10 bands compared with the control group. The bands included 219 non-redundant cellular proteins that interact with M protein, which were identified by LC-MS/MS with high confidence. The gene ontology and Kyoto encyclopedia of genes and genomes (KEGG) pathway bioinformatic analyses indicated that the identified proteins could be assigned to several different subcellular locations and functional classes. Functional analysis of the interactome profile highlighted cellular pathways associated with protein translation, infectious disease, and signal transduction. Two interested cellular proteins—nuclear factor of activated T cells 45 kDa (NF45) and proliferating cell nuclear antigen (PCNA)—that could interact with M protein were validated by Co-IP and confocal analyses.

**Conclusions:**

The interactome data between PRRSV M protein and cellular proteins were identified and contribute to the understanding of the roles of M protein in the replication and pathogenesis of PRRSV. The interactome of M protein will aid studies of virus/host interactions and provide means to decrease the threat of PRRSV to the swine industry in the future.

**Electronic supplementary material:**

The online version of this article (doi:10.1186/s12985-017-0700-1) contains supplementary material, which is available to authorized users.

## Background

Porcine reproductive and respiratory syndrome virus (PRRSV) is the etiologic agent of porcine reproductive and respiratory syndrome (PRRS) [1–4], an economically devastating pandemic disease of swine. PRRS is typically characterized by severe reproductive failure in sows and respiratory disorders in pigs of all ages [5, 6]. The disease is now found in most pig-producing countries and affects the swine industry and food safety worldwide [7–9]. In particular, the emergence of highly pathogenic PRRSVs (HP-PRRSVs) in China and Vietnam in 2006 [10–14] and their rapid spread to several neighboring Asian countries [15] has raised concerns that a new pathogenic PRRSVs could spread throughout the world, posing a substantial threat to the global agricultural community [16–18].

PRRSV is an enveloped, single-stranded, positive-sense RNA virus belonging to the order *Nidovirales*, family *Arteriviridae*, and genus *Arterivirus* [3, 19]. The viral genome is approximately 15 kb in length and encodes at least 10 open reading frames (ORFs), comprising of ORF1a, ORF1b, ORF2a, ORF2b, ORFs3–7 and the recently discovered ORF5a [20, 21]. ORF1a and ORF1b encode viral replicase polyproteins, which are proteolytically processed by virally encoded proteinases into 14 mature nonstructural proteins and the newly discovered transframe fusion (TF) in the NSP2-coding region [22–24]. The rest of the ORFs of PRRSV encode eight structural proteins: GP2, E, GP3, GP4, GP5, M, N, and ORF5a [20, 25, 26]. The M protein, an 18 to 19 kDa class III membrane protein, is unglycosylated and the most conserved structural protein of arteriviruses and PRRSV [27, 28]. The M protein is a key target for PRRSV neutralization [29]. A bacillus Calmette-Guérin vaccine strain of *Mycobacterium bovisbacille* expressing the M protein successfully induced the development of M protein neutralizing antibodies in mice, further indicating that the M protein contains neutralizing epitopes [30]. Co-expression of GP5 and M protein as heterodimers significantly improves the potency of PRRSV DNA vaccination [31]. The M protein and GP5 are found as a disulfide -linked heterodimer in the virion, which is essential for the infectivity of arteriviruses [32, 33]. The M protein and the M/GP5 complex contribute to PRRSV attachment to a heparinlike receptor on pulmonary alveolar macrophages (PAMs) [34]. The M/GP5 complex was identified as a ligand for sialoadhesin, which is involved in the entry process of PRRSV in to PAMs [35, 36]. These findings reveal that the M protein is involved in not only PRRSV infection and immunity but also the entry process of the pathogen. However, the molecular mechanisms of its involvement in these functions have not been elucidated clearly.

We used the co-immunoprecipitation (Co-IP) technique coupled with LC-MS/MS and bioinformatics analysis to screen and analyze host cellular proteins interaction with PRRSV M protein. An interactome profile of M protein was generated to understand the mechanism of PRRSV infection and immunity.

## Methods

### Cells, virus, and plasmid

The MARC-145 and 293 T cell lines were cultured in Dulbecco’s modified eagle medium (DMEM) (Gibco BRL, Gaithersburg, MD, USA) containing 10% fetal bovine serum (FBS) (Hyclone Laboratories, Inc., South Logan, UT) at 37 °C, with 5% CO_2_. The hybridoma cell line named 3 F7 secreting PRRSV M protein mAb was prepared by our lab [37]. The 3 F7 monoclonal antibody subclass was IgG1. The IFA titer of the 3 F7 culture supernatant was 1:512, and the Western blot titer of it was at least 1:512. The most important is that the binding of the 3 F7 to PRRSV could be blocked by the anti-serum to PRRSV in blocking ELISA. A HP-PRRS vaccine strain HuN4-F112 was obtained by culturing its parent strain, HP-PRRSV HuN4 [12, 13], with MARC-145 cells for 112 passages [38]. The 5th-passage HuN4 (HuN4-F5) used in animal’s challenge [38] was used in CO-IP together with HuN4-F112. The eukaryotic expression vector pCAGGS-Flag-HuN4-F112-M was maintained in our lab.

### Purification of M protein mAb

BALB/c mice aged 12 weeks (from the Laboratory Animal Center of Harbin Veterinary Research Institute, CAAS) were primed with Freund’s incomplete adjuvant (Sigma, St. Louis, MO, USA) and administered an intraperitoneal injection of 1-3 × 10^6^ hybridoma cell 3 days later. Ascitic fluids were collected using syringes when abdominal distension became marked. The mice were euthanized after three collections. The M protein mAb was purified by Protein G resin (GenScript, Nanjing, China) according to the manufacturer’s instructions.

### Plasmid construction

A 4-week-old SPF landrace piglet was obtained from the Laboratory Animal Center of Harbin Veterinary Research Institute, CAAS. The piglet was euthanized, and its pulmonary alveolar macrophages were collected according to a previously described method [39]. The ORFs of NF45 and PCNA were amplified from the total RNA of PAMs by RT-PCR using the designed primers based on the sequences available from GenBank (XM_005663409.1, NF45; GQ913657.1, PCNA). The reverse transcriptions were performed using AMV reverse transcriptase (Takara, Dalian, China) in a reaction system with a total volume of 20 μL. The ORF6 gene of PRRSV was amplified by PCR using pCAGGS-Flag-HuN4-F112-M as the template. The pCMV-HA-NF45/PCNA, pCAGGS-Flag-NF45/PCNA and pCMV-HA-M plasmids were constructed by conventional techniques. All the primers used in this study are listed in Table 1.

**Table 1:** Primers for construction of enkaryotic expression vectors

Primer	Sequence (5′-3′)	Use
HA-NF45-F	CCGGAATTCGGATGAGGGGTGACAGAG	Construction of pCMV-HA-NF45
HA-NF45-R	CCGCTCGAG **TCA**CTCCTGAGTTTCCATG	
HA-PCNA-F	CGCGTCGACCATGTTCGAGGCGCGCC	Construction of pCMV-HA-PCNA
HA-PCNA-R	CCGCTCGAGCTAAGACCCTTCTTCATCTTCG	
HA-M-F	CCGGAATTCGGATGGGGTCGTCTCTAGACG	Construction of pCMV-HA-M
HA-M-R	CCGCTCGAG **TTA**TTTGGCATATTTAACAAGG	
Flag-NF45-F	CCGGAATTCGGATGAGGGGTGACAGAG	Construction of pCAGGS-Flag-NF45
Flag-NF45-R	CCGCTCGAGCACTCCTGAGTTTCCATG	
Flag-PCNA-F	GCCGAGCTCAATGTTCGAGGCGCG	Construction of pCAGGS-Flag-PCNA
Flag-PCNA-R	CCGCTCGAGTAAGACCCTTCTTCATCTTCG	

Note: F denotes forward PCR primer, R denotes reverse PCR primer; restriction sites are underlined; the termination codons are bold

### Detecting the expression of M protein

MARC-145 cells in a 60-mm dish were infected with the HuN4-F112 at an MOI of 0.1. The cells were collected at different time points (12 to 84 h post-infection). The samples were subjected to Western blot with anti-M protein mAb. The assay was repeated in triplicate.

### Co-Immunoprecipitation

HuN4-F112/HuN4-F5 infected MARC-145 cells and uninfected MARC-145 cells were lysed in NP-40 lysis buffer (Beyotime, Nanjing, China) containing 1 mM phenylmethylsulfonyl fluoride (PMSF) (Beyotime, Nanjing, China) and 1% protease inhibitor cocktail (Sigma, St. Louis, MO, USA) by incubation at 4 °C on a shaker for 30 min, followed by centrifugation at 12,000 × g for 20 min. Clarified extracts were precleared with protein G beads for 1 h. A total of 1 mL of each supernatant at a final concentration of 5 mg/mL was precipitated with anti-M protein mAb 3 F7 in conjunction with protein G resin and incubated with gentle rocking overnight at 4 °C. The beads were washed five times with PBS and boiled with 1 × SDS loading buffer for 5 min, followed by SDS-PAGE and Coomassie brilliant blue staining or Western blot. 293 T cells were transfected with the constructed plasmids as described above to verify the interaction between M protein and host proteins. Cells co-transfected with empty vector pCMV-HA or pCAGGS-Flag served as controls. The assay was repeated in triplicate.

### Coomassie blue staining and mass spectrometric identification of proteins

The immunoprecipitated proteins were separated by electrophoresis on 5% and 12% SDS-PAGE gels and the separation gel was stained using Coomassie brilliant blue for Mass Spectrometry. All distinct bands in the lane of HuN4-F112 infection group and the gel at parallel areas in the lane of the control group were excised and subjected to LC-MS/MS. Briefly, gel pieces were distained with 30% acetonitrile/100 mM NH_4_HCO_3_ and freeze-dried. The gel pieces were reduced with 100 mM of DTT (56 °C, 30 min), followed by alkylation with 200 mM iodoacetamide (in the dark, 25 °C, 20 min). The gels were incubated with 100 mM NH_4_HCO_3_ and shrunk with acetonitrile again, and incubated with trypsin (2.5-10 ng/μL) for 20 h at 37 °C. Peptides were extracted with 60% acetonitrile/0.1% TFA. Peptides were separated using a nano-flow HPLC (LTQ VELOS, Thermo Finnigan, San Jose, CA, USA).

### Bioinformatics analysis

The functional annotation and classification of all the proteins were determined using Blast2GO program [40] against the non-redundant protein database (nr) at NCBI and the KEGG pathway database [41]. The protein-protein interact network was performed using Cytoscape software [42].

### Western blot analysis

Protein samples were separated by 12% SDS-PAGE and then transferred onto PVDF membranes (Millipore, Bedford, MA, USA). The membranes were incubated with anti-HA mAb, anti-Flag mAb, and anti-M mAb, respectively. After the membranes were rinsed with PBST, each membrane was treated with DyLight 800-Goat Anti-Mouse IgG (H + L) as the secondary antibody. The proteins were visualized by scanning the membranes with a LI-COR Odyssey infrared image system (LI-COR Biosciences, Lincoln, NE, USA).

### Confocal imaging

293 T cells were cotransfected with pCAGGS-Flag-NF45/PCNA (0.5 μg) and pCMV-HA-M (0.5 μg) in a 35-mm dish. After 48 h of incubation, transfected cells were fixed with 4% paraformaldehyde in PBS for 30 min and permeabilized with 0.1% Triton X-100 for 15 min. The cells were incubated with anti-HA mAb and anti-Flag pAb for 1 h. The cells were then incubated with goat anti-mouse IgG -FITC (F2012; Sigma) and goat anti-rabbit IgG-TRITC antibodies. Cells were stained with DAPI for 5 min and examined with a Leica SP2 confocal system (Leica Microsystems, Wetzlar, Hessen, Germany). The assay was repeated in triplicate.

## Results

### The expression of M protein upon PRRSV infection

**Fig. 1:**
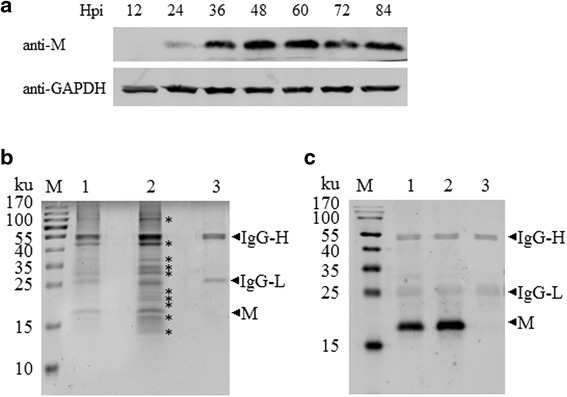
Identification of the cellular proteins that interact with PRRSV M protein by co-immunoprecipitation (Co-IP). **a** Cell lysates from HP-PRRSV strain HuN4-F112-infected MARC-145 cells at different time points were subjected to Western blot with anti-M protein mAb 3 F7 and anti-GAPDH mAb. **b** Cell lysates from HuN4-F112/HuN4-F5- or mock-infected MARC-145 cells were immunoprecipitated with anti-M protein mAb 3 F7. The three lanes (lane 1, lane 2, and lane 3) stand for HuN4-F5 infected group, HuN4-F112 infected group, and control group, respectively. The immunoprecipitated proteins were separated by 12% SDS-PAGE and visualized by Coomassie brilliant blue staining. The asterisks (*) show the differential protein bands between HuN4-F112- or mock-infected MARC-145 cells. **c** The experimental procedure was as the same as above, but the cell lysates were subjected to Western blot with anti-M protein mAb 3 F7

MARC-145 cells were infected with the HuN4-F112 and collected at 12 h to 84 h post-infection to detect the expression of M protein arising from PRRSV infection. Samples were subjected to Western blot analysis with anti-M protein mAb. Expression of GAPDH served as an internal reference. The expression level of M protein increased during PRRSV infection and reached a peak between 48 and 60 h post-infection (Fig. 1a). We collected samples at 48–60 h post-infection for subsequent interactome analyses.

### Identification of host cellular proteins that interact with PRRSV M protein

MARC-145 cells were infected with HuN4-F112/HuN4-F5 at an MOI of 0.1 to efficiently precipitate M protein and subsequently identify host proteins that interact with M protein. Infected cells were harvested at 48–60 h post-infection and immunoprecipitated with M protein mAb 3 F7. The immunoprecipitated proteins were resolved using 12% SDS-PAGE and visualized by Coomassie brilliant blue staining (Fig. 1b) and Western blot with 3 F7 (Fig. 1c). At least 10 additional bands of proteins specifically precipitated from HuN4-F112 infected cells compared with the control group (Fig. 1b). 3011 cellular proteins were identified by LC-MS/MS analysis of the 10 protein bands coming from the HuN4-F112 infection group. Of these, 219 proteins had a high confidence (Unique Peptide ≥ 2). A summary of the proteins that interact with M protein at 48–60 h following PRRSV infection are given in (Additional file 1: Table S1), with the UniquePepCount and CoverPercent of each protein.

### Functional analyses of identified proteins

**Fig. 2:**
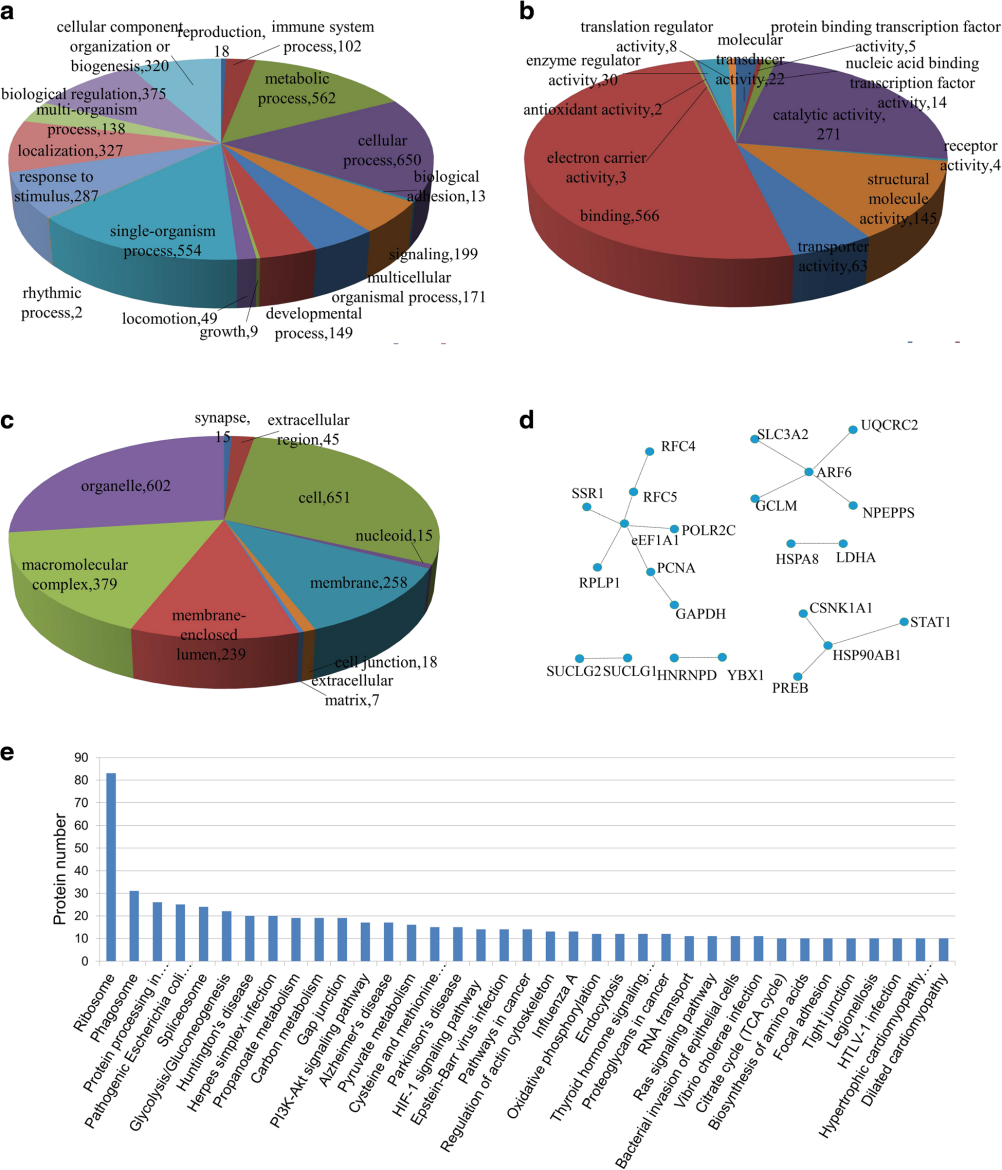
The annotation of proteins interacting with PRRSV M protein using Gene Ontology, the interaction network, and the enriched KEGG pathways. **a** Biological process. **b** Cellular components. **c** Molecular function. **d** Interaction network. **e** Classification of the enriched KEGG pathways of the cellular proteins interacting with PRRSV M protein

All of the identified proteins were assigned for bioinformatic analyses to gain functional insights into the interactome of M protein. Three main types of annotations, including biological processes, cellular components and molecular functions, were obtained from the gene ontology (GO) consortium website (Fig. 2a–c). Subclasses associated with cellular process (16.57%), metabolic process (14.32%), single-organism process (14.12%), biological regulation (9.56%), localization (8.34%) and cellular component organization or biogenesis (8.16%) were enriched in the biological process category (Fig. 2a). The most enriched subclasses in the cellular components included cell (29.21%), organelle (27%), macromolecular complex (17%), membrane (11.57%) and membrane-enclosed lumen (10.72%) (Fig. 2c). The enrichments based on molecular function were binding (49.96%), catalytic activity (23.9%) and structural molecule activity (12.8%) (Fig. 2b). A more detailed summary containing the GO annotation for individual proteins is provided in (Additional file 2: Table S2).

The interactions between the differentially expressed proteins and other proteins were determined by querying the IntAct data base according to differentially expressed proteins’ Gene Symbol. The interaction network of the cellular proteins interacting with M protein were drawn using the CytoScape software (Fig. 2d).

Analysis of the infection network based on the KEGG revealed an enrichment of 219 pathways (Additional file 3: Table S3). The more prominent pathways were involved in ribosome (83 proteins), phagosome (31 proteins), protein processing in endoplasmic reticulum (26 proteins), pathogenic *Escherichia coli* infection (25 proteins), spliceosome (24 proteins) and glycolysis/gluconeogenesis (22 proteins) (Fig. 2e and Additional file 3: Table S3).

### Validation of the proteins that interact with the M protein of PRRSV by CO-IP

**Fig. 3:**
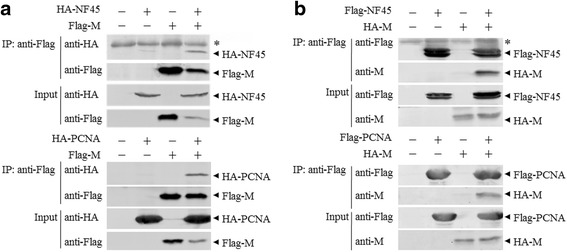
Confirmation of the interaction of PRRSV M protein with NF45 and PCNA by CO-IP. The interaction of M protein and exogenous NF45 and PCNA. 293 T cells were co-transfected with 5 μg of the indicated plasmids in 60-mm dishes. Cell lysates were prepared at 36–48 h after transfection and the proteins were immunoprecipitated with anti-Flag mAb. Proteins in cell lysates (input) and immunoprecipitated samples were detected with the antibodies against Flag, HA, and M by Western blot. The asterisk (*) indicates IgG (Flag mAb) heavy chains. **a** NF45 and PCNA were immunoprecipitated by M protein. **b** M protein was immunoprecipitated by NF45 and PCNA

The total RNA of PAMs was extracted and the ORFs of two interested protein (NF45 and PCNA) were amplified by RT-PCR. PCR products were cloned into the pCMV-HA/pCAGGS-Flag vectors and confirmed by sequencing. A pCMV-HA-HuN4-F112-M vector was constructed in the same way. After the 293 T cells were transfected with pCAGGS-Flag-M and pCMV-HA-NF45/PCNA or with pCMV-HA-M and pCAGGS-Flag-NF45/PCNA, Co-IP was performed with ANTI-FLAG® M2 Affinity Gel (Sigma, St. Louis, MO, USA). The immune complexes were resolved by 12% SDS-PAGE and probed for the presence of NF45/PCNA or M protein using anti-HA mAb and anti-M protein mAb. Both NF45 and PCNA were readily detected in the presence of M protein, but not in the presence of empty vector (Fig. 3a). M protein was only detected in the presence of NF45 or PCNA (Fig. 3b). These results confirmed that PRRSV M protein was able to interact with the overexpressed proteins NF45 and PCNA.

### Confocal analyses of M protein and NF45/PCNA

**Fig. 4:**
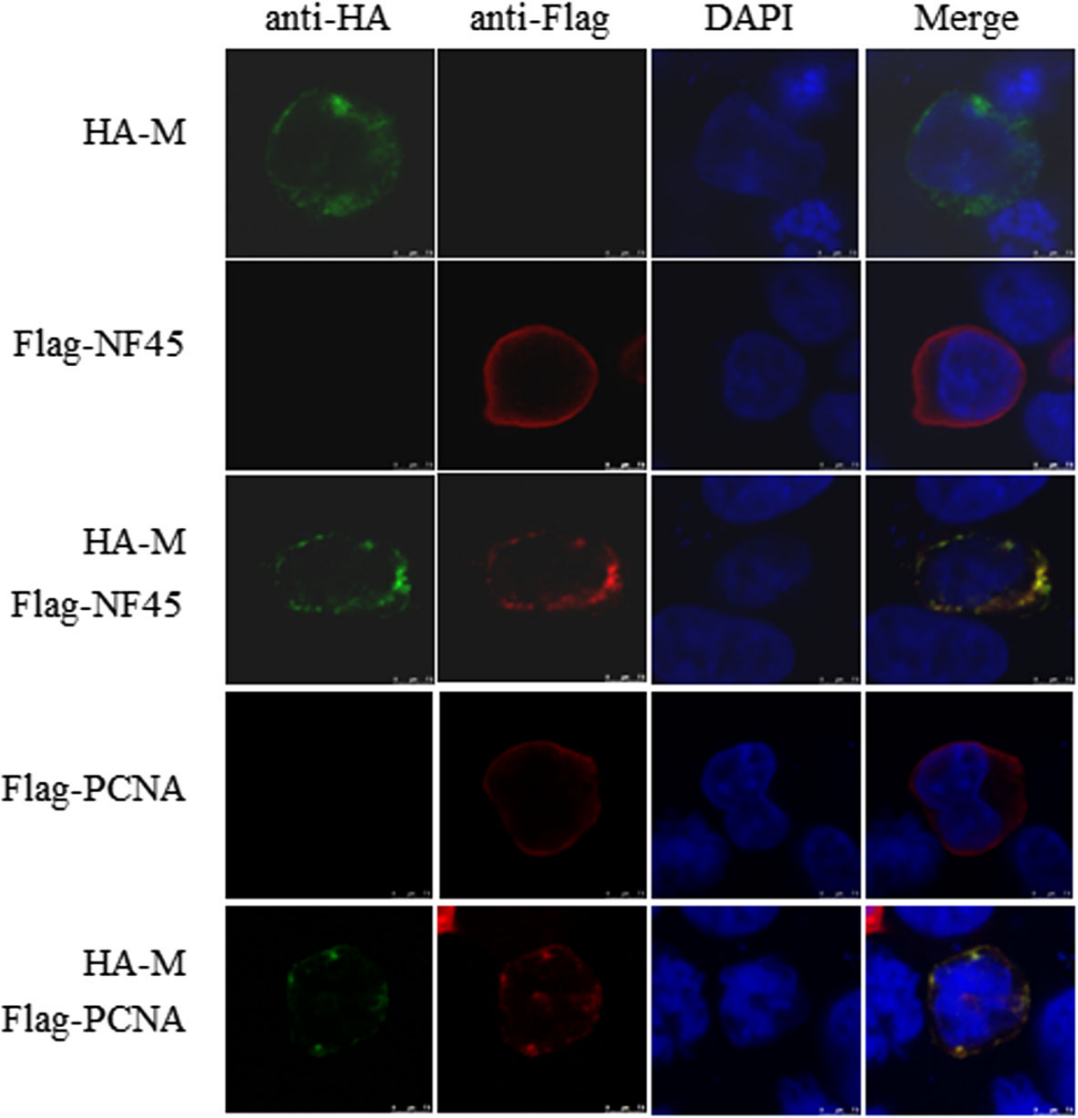
Colocalization of M protein with NF45 and PCNA. 293 T cells were cotransfected with HA-M and Flag-NF45/PCNA. Cells were fixed at 48 h and subjected to indirect immunofluorescence to detect HA-M (green) and Flag-NF45/PCNA (red) with mouse anti-HA and rabbit anti-Flag antibodies. The position of the nucleus is indicated by DAPI (blue) staining in the merged image

To examine the colocalization of M protein with NF45 or PCNA, 293 T cells were co-transfected with plasmids expressing HA-M and Flag-NF45/PCNA proteins and the subcellular localization of M protein and NF45 or PCNA were examined by confocal microscopy (Fig. 4). Both HA-M protein and Flag-NF45/PCNA were distributed throughout the cytoplasm, and M protein colocalized with NF45 or PCNA. This finding confirms that M protein interacts with exogenous NF45 or PCNA in 293 T cells.

## Discussion

PRRSV causes persistent infection and immunological tolerance in pigs [43], but the specific molecular mechanisms of these effects have not been absolutely elucidated. The virus proteins carry out some functions that depend on interaction with the host cellular proteins, so it is necessary to explore the mechanisms associated with viral pathogenesis and host anti-virus response using a protein interactions approach.

The M protein encoded by ORF6 is an unglycosylated membrane protein of 18–19 kDa [27, 43]. The M protein is important in virus assembly and budding [44] and is linked to GP5 as heterodimers via a disulfide bond at the N-terminal ectodomains [27, 45]. The M/GP5 complex could combine sialoadhesin, which is involved in the entry process of PRRSV in to PAM [35, 36]. Investigating the interactome profile of M protein with the host cellular proteins is very valuable because PRRSV M protein has important functions associated with viral entry and replication. In the present study, the HP-PRRSV vaccine strain HuN4-F112 was used to further investigate the direct and indirect interaction of cellular proteins with M protein in PRRSV-infected MARC-145 cells. This viral strain was chosen because the vaccine strain HuN4-F112 was more adaptive to the MARC-145 cells than other HP-PRRSVs including HuN4-F5, which was useful to screen more host cellular proteins. This method can present the native protein conformation during virus replication and explore the cellular proteins that interact directly or indirectly with M protein in the presence of other viral proteins. These interactions are easily missed using the classical method of Co-IP of a single protein with host cells [46, 47].

In this study, 219 host cellular proteins that interact with M protein were identified in HuN4-F112-infected cells with high confidence by Co-IP and LC-MS/MS. We used bioinformatic analysis to comprehensively evaluate and characterize the identified proteins to further explore the biological significance of the interaction between M protein and host cellular proteins. The results implicate a large number of host cellular proteins that were related to the ribosome, protein processing in the endoplasmic reticulum, spliceosome, phagosome, pathogenic *Escherichia coli* infection, and glycolysis pathways. Of these, the first three pathways were related to protein translation and it is reasonable to find these translation pathways were enriched during the PRRSV infection. The translation process is initiated after virus entry and release of the viral genome into the cytoplasm. The PRRSV first translates its two replicase proteins coded by ORF1a and ORF1b, by employing the host translation system, to yield the polyprotein precursors pp1a and pp1ab [22, 48, 49]. Our data suggest that M protein could interact with proteins related to protein translation. The GO annotations of the host cellular proteins that interacted with M protein indicated they were located on the membrane and had binding and catalytic activities, and we inferred that M protein could combine with membrane proteins. Previous studies showed that heparin interacted with the virus and reduced infection of PAM by up to 92% or 88% for the American and European types of PRRSV, respectively [34]. Heparinase treatment of PAM resulted in a significant reduction of the infection. The structural M protein and the M/GP5 complex were verified to contribute to PRRSV attachment on a heparinlike receptor on PAM using heparin-affinity chromatography and SDS-PAGE [34]. These results further suggested that M protein could combine with membrane proteins. The interaction network of differentially expressed proteins is shown in Fig. 2d, which identified over 20 proteins with six small dispersed protein networks. The networks of proteins interacting with PRRSV M protein were poorly understood until now. There are three proteins located in the center, eukaryotic elongation factor 1A (eEF1A), ADP-ribosylation factor (ARF) 6, and the cellular chaperone HSP90AB1. The eEF1A is one of the most abundant protein synthesis factors, and constitutes 1% to 4% of the total soluble proteins in actively dividing cells [50, 51]. eEF1A takes part in viral transcription, translation and assembly as a cofactor for many viruses, including tombusvirus (TBSV) [52] and human immunodeficiency type 1 (HIV-1) [53]. Moreover, eEF1A interacts with the NS5A protein and inhibits the growth of classical swine fever virus (CSFV) [54]. ARFs are 21-kDa GTP-binding proteins that belong to a group of ras-related small GTPases that regulate various events associated with membrane trafficking. The ARFs constitute a family of gene products composed of six ARF proteins and nine ARF-like proteins. The ARFs are divided into three classes based on size and amino acid identity: ARFs 1, 2, 3 and ARFs 4, 5 constitute classes I and II, respectively, with ARF6 belonging to class III. In fact, ARF6 is the only member of the Ras-related ARF family of small GTPases that affects cell-surface dynamics, thereby regulating plasma membrane/endosome trafficking and cortical actin reorganization [55]. HIV-1 requires ARF6-mediated membrane dynamics to efficiently enter and infect T lymphocytes [56]. HSP90AB1 is an abundant, highly conserved cellular chaperone that functions as a key component of a multiprotein chaperone complex. These complex includes Cdc37 and several other proteins that regulate folding, maturation, stabilization, and renaturation of a select group of target proteins [57, 58]. A previous study has demonstrated that hepatitis B virus polymerase suppresses NF-κB signaling by inhibiting the activity of IKKs via interaction with HSP90AB1 [59]. All of these findings show that these three protein participated in virus replication and innate immunity, which may also play a role in PRRSV life cycle via interaction with M protein.

A proportion of proteins were shown to be associated with the infectious disease (Fig. 3 and Additional file 3: Table S3). These results implicate that like others pathogens, PRRSV may exploit similar host cellular components and share a common or similar pathogenesis. Thus the research on other pathogens could be useful in the study of PRRSV pathogenesis.

We selected two novel proteins from the 219 cellular proteins that interact with M protein, namely NF45 and PCNA, and the interactions between PRRSV M protein and porcine NF45 or PCNA were further confirmed by Co-IP (Fig. 4). NF45 is a versatile nuclear protein that associates with various factors in multifunctional complexes involved in mitosis, microRNA biogenesis [60], interleukin 2 (IL-2) production [61], IRES-dependent translational control [62], and cellular inhibitor of apoptosis protein 1 (cIAP1)-mediated antiapoptosis [63]. Recent observations suggest that NF45 and its heterodimer NF90 are significantly involved in the replication process of several different RNA viruses. Both NF45 and NF90 were indicated to be part of viral replication machineries and suggested to part of the regulation of viral translation and RNA replication for two Flaviviridae family members, bovine viral diarrhea virus (BVDV) and hepatitis C virus (HCV) [64–66]. NF45 interacts with viral proteins of infectious bursal disease virus and inhibits viral replication [67]. We identified another protein-proliferating cell nuclear antigen (PCNA) among the proteins identified from the interactome profile of M protein (Fig. 4). PCNA is a member of the sliding clamp family of DNA-replication accessory proteins. Their functions are critical to processes such as cell cycle control, chromatin remodeling, gene expression, apoptosis, and DNA repair [68–71]. PCNA is a homo episomal trimer in most organisms, with three subunits that adopt a doughnut-shaped structure in a head-to-tail arrangement. This toroidal structure is highly conserved in protozoa, humans, yeasts and plants [72–75]. Ubiquitylation of PCNA participates directly in the meiotic process and the diversification of the Ig locus through class-switch recombination and somatic hypermutation [76]. PCNA was identified as an H5N1 PA-host interacting protein in chicken cells [77]. All seven viral replication proteins of herpes simplex virus were enriched on the viral genome, along with cellular PCNA [78]. PCNA was recruited by LANA to the Kaposi’s Sarcoma-associated herpesvirus genome via Bub1 to initiate viral replication during the cell division S phase [79]. We inferred that PCNA was involved in the replication of many viral genomes. Both NF45 and PCNA are found predominantly in the nucleus, and they may interact with M protein in the cytoplasm after nuclear export.

The interaction between virus and a host cell is not only the process that the virus replicates and cells releases progeny virus using host cell and viral proteins after breaking through multi-level barriers, but also the process that host cell resists virus invasion or self-sacrifice to clear the virus. These interactions ultimately results in changes in protein expression patterns, which influence normal physiology function of host cells and ultimately determines the processes and results of viral infection. The M protein has important biological functions during PRRSV infection and immunity. Our findings about the proteins that interact with the M protein provide scientific clues for understanding virus molecular pathogenesis and control.

These findings not only generate new sight on the cellular defense mechanism against PRRSV infections, but also provide a new view on PRRSV participating in cell cycle control.

## Conclusions

In the present study, 219 host cellular proteins that interact with the M protein in PRRSV-infected cells were identified with high confidence using a HP-PRRSV vaccine by a Co-IP and LC/MS-MS coupled method. The identified proteins were assigned to different subcellular locations and functional classes according to the GO annotation and enriched KEGG pathway analysis. An interactome profile of M protein with the host cellular proteins was drawn to gain a functional insight into the host-virus proteins interaction.

## Additional files

Additional file 1:
**Table S1.** The list of the proteins interacting with the PRRSV M protein. (XLS 61 kb)
Additional file 2:
**Table S2.** The annotation of proteins interacting with M protein during PRRSV infection using Gene Ontology. (XLS 2143 kb)
Additional file 3:
**Table S3.** The list of the enriched KEGG Pathways of the PRRSV M protein interacting proteins. (XLS 277 kb)

## Abbreviations

DTT: Dithiothreitol
GAPDH: Glyceraldehyde-3-phosphate dehydrogenase
LC-MS/MS: Liquid Chromatography-Mass Spectrometry/Mass Spectrometry
MOI: Multiplicity of infection
NF45: Nuclear factor of activated T cells 45 kDa
PCNA: Proliferating cell nuclear antigen
RT-PCR: Reverse transcriptase-polymerase chain reaction
SDS-PAGE: Sodium dodecyl sulfate polyacrylamide gel electrophoresis
TFA: Trifluoroacetic acid.
